# AI-based atomic force microscopy image analysis allows to predict electrochemical impedance spectra of defects in tethered bilayer membranes

**DOI:** 10.1038/s41598-022-04853-4

**Published:** 2022-01-21

**Authors:** Tomas Raila, Tadas Penkauskas, Filipas Ambrulevičius, Marija Jankunec, Tadas Meškauskas, Gintaras Valinčius

**Affiliations:** 1 Institute of Computer Science, Vilnius University, Didlaukio 47, 08303 Vilnius, Lithuania; 2 Institute of Biochemistry, Life Sciences Center, Vilnius University, Saulėtekio 7, 10257 Vilnius, Lithuania

**Keywords:** Biophysical chemistry, Ion channels, Lipids, Structural biology, Membrane biophysics, Membrane structure and assembly, Permeation and transport, Electrochemistry, Surface chemistry, Applied mathematics, Computational science

## Abstract

Atomic force microscopy (AFM) image analysis of supported bilayers, such as tethered bilayer membranes (tBLMs) can reveal the nature of the membrane damage by pore-forming proteins and predict the electrochemical impedance spectroscopy (EIS) response of such objects. However, automated analysis involving pore detection in such images is often non-trivial and can require AI-based object detection techniques. The specific object-detection algorithm we used to determine the defect coordinates in real AFM images was a convolutional neural network (CNN). Defect coordinates allow to predict the EIS response of tBLMs populated by the pore-forming toxins using finite element analysis (FEA) modeling. We tested if the accuracy of the CNN algorithm affected the EIS spectral features sensitive to defect densities and other physical parameters of tBLMs. We found that the EIS spectra can be predicted sufficiently well, however, systematic errors of characteristic spectral points were observed and need to be taken into account. Importantly, the comparison of predicted EIS curves with experimental ones allowed to estimate important physical parameters of tBLMs such as the specific resistance of submembrane reservoir. This reservoir separates phospholipid bilayer from the solid support. We found that the specific resistance of the reservoir amounts to $10^{4.25 \pm 0.10}$ $\Omega \cdot cm$ which is approximately two orders of a magnitude higher compared to the specific resistance of the buffer bathing tBLMs studied in this work. We hypothesize that such effect may be related in part due to decreased concentration of ionic carriers in the submembrane due to decreased relative dielectric permittivity in this region.

## Introduction

Atomic Force Microscopy (AFM) is increasingly used for studying interaction of lipid bilayers with proteins including pore-forming toxins (PFTs) and membrane disrupting peptides^1–3^. AFM is capable of detecting insertion of proteins, heterogeneous distribution of proteins in membranes^2^ in phase separated membranes^3^, formation of rings of PFTs^1^ and other structural details important to understand how membrane protein interact with cell membranes.

While providing nanoscale-level structural details of reconstituted PFT’s and peptides in membranes, AFM does not directly access function of these proteins, neither it can predict the extent of dielectric damage by PFTs and peptide. Such information is important in establishing fundamental relation between structure and function of biological systems.

Because of evident reasons the AFM studies of membrane proteins are performed using solid supported phospholipid bilayers^4^. In case the electrical conductance data reflecting functional effects of PFTs or peptides on membranes is sought the tethered bilayer systems are used^5,6^. Also, both techniques, AFM and EIS, are used simultaneously or in parallel to characterize structure and function of PFTs in membranes^7–10^.

The electrochemical impedance spectroscopy (EIS) is a method of choice for detailed studies of electrical effects of PFTs in membranes. The EIS allows accessing the dielectric properties and conductance data of tBLMs (tethered bilayer membranes), and in some cases though not being structural method per se, provides insights into lateral distribution of defects in membranes^8–10^. So far, however, there were no attempts to quantitatively relate structural data obtained by AFM and the membrane conductance data measured by EIS, even though experimental capabilities to apply both techniques on the same membrane samples are straightforward. Such comparative measurements would be of great value in studying function of both single and multiple ensembles of membrane damaging protein entities as well as in developing precision biosensors based on tBLMs^11,12^.

Recently, significant progress has been made in the development of EIS data analysis of solid supported (tethered) phospholipid membranes^8–10,13^. In particular, the theoretical analysis demonstrated that the amount of reconstituted protein pores per surface area can be retrieved from the EIS spectral data. Nevertheless, such theoretical approaches, strictly speaking, should be verified by using data from the independent structural techniques such as AFM.

The objective of current study is to explore the possibility to predict the electrochemical impedance spectra from the AFM images of membranes with reconstituted PFTs. The AFM technique allows to detect PFT entities which appear on tBLM surface upon exposure of bilayer to the protein solution. The coordinates of these entities may be measured, and the finite element analysis (FEA) can be applied to model EIS response of such supported membranes. The comparison of predicted and experimental EIS curves obtained from the same sample would allow (1) to independently verify the applicability of FEA approach to theoretically predict EIS spectra developed earlier^9,10^ on real, AFM imaged surfaces, (2) to precisely evaluate the physical parameters of supported bilayer membranes, among which the specific resistance of submembrane reservoir separating bilayer from the solid support is of upmost importance. This parameter is strongly correlated with the density of PFT defects in tBLMs^13,14^, therefore, independent verification by AFM can resolve the ambiguities related to such correlation.

Typically, only a tiny patch compared to a whole surface area is interrogated by the AFM technique. To establish representative defect densities and their distribution patterns, the sufficiently large areas, in our case, containing hundreds and thousands of defects must by tested. The determination of coordinates of large defect ensembles is a highly time consuming process. To overcome such and similar problems automated algorithms can be applied for AFM image analysis.

Typically, the features of different shapes in AFM images are detected via particle or grain analysis based on edge detection. In the majority of cases, a pre-processing takes place to make it easier to measure and observe the features that have been measured^15^. AFM images are always affected by the geometry of a tip and external noise that disturb image features. Although basic image segmentation approaches work well for good-quality image data containing clear and easily distinguishable objects, analysis of noisy, low-resolution or otherwise degraded images requires more sophisticated methods. An important factor is the scarcity of such image data which limits the possibilities of applying machine learning or deep learning methods in a practical way. In some cases researchers still resort to manual work of annotating and quantifying objects of interest in microscopy images^7,16^.

Despite the difficulties associated with the automated analysis of AFM images, substantial progress has been recently made in developing practical solutions for certain types of such problems. Meng et al.^17^ presented an algorithm based on local adaptive Canny edge detection and circular Hough transform which is suitable for recognizing particles in scanning electron microscope (SEM) or transmission electron microscope (TEM) images. Another study conducted by Venkataraman et al.^18^ showed that rotavirus particles in AFM images can be detected by applying a series of image pre-processing, segmentation and morphological operations. Marsh et al.^19^ proposed the Hessian blob algorithm for detecting biomolecules in AFM images and showed its superiority against the threshold and watershed image segmentation algorithms. Other recent studies also showed that deep learning techniques can be successfully applied to detect complex-shaped objects in microscopy images. Sotres et al.^20^ used the YOLOv3 object detection model and a Siamese neural network to determine the locations of DNA molecules in AFM images and identify the same molecule in different images. Okunev et al.^21^ applied a Cascade Mask-RCNN neural network to detect metal nanoparticles in scanning tunneling microscopy (STM) images. In both of these cases the researchers used precision and recall metrics to measure the performance of the proposed models. One more study by Sundstrom et al.^22^ involved a supervised learning approach of estimating lengths of DNA molecules in AFM images. A software tool for the automated biomolecule tracing in AFM data (TopoStats) was also recently developed and presented by Beton et al.^23^

In this study we investigate the problem of automated detection of membrane bound PFTs in AFM images. Performing this task with adequate accuracy is of practical importance, as the determined coordinates would allow to theoretically calculate EIS spectral features and to compare those features with the experimental EIS data. In addition to applying and testing one of the popular computer vision techniques—convolutional neural network, we present a method for generating synthetic defect sets which resemble detection results of varying accuracy, similar to those obtained by using an actual object detection model. Such datasets are used to perform FEA modeling of EIS spectra and examine the relationship between defect detection accuracy and corresponding variations of EIS spectral features. By doing so we address the question—whether there is some minimal requirement for the precision of the AI based image processing algorithm so that the EIS spectra prediction would fall into acceptable range of uncertainty?

## Methods

### AFM imaging

AFM image data was obtained by measuring three separate tBLM membrane cells. Assembled tethered lipid bilayers were incubated for 30 min with vaginolysin (VLY). Aliquot of a toxin was added to the cell, so that final concentration of VLY was ${1}\,\hbox {nM}$. After incubation, cell was washed with 10 mL of phosphate buffer $\hbox {pH} {7.1}$ to remove any unbound protein debris, and disassembled under water. AFM imaging was carried out in aqueous environment. More detailed description of experimental settings can be found elsewhere^10^.

For each cell a surface patch of ${6}\,\upmu \hbox {m} \times {6}\,\upmu \hbox {m}$ was scanned by capturing one ${2}\,\upmu \hbox {m} \times {2}\,\upmu \hbox {m}$ fragment at a time. Each fragment was imaged with $512 \times 512$ resolution, thus the overall stitched image consisting of $3 \times 3$ fragments had $1536 \times 1536$ resolution. Each image fragment was manually annotated by marking center coordinates (X and Y) of each defect visible in the image. Image fragment sets of each cell were partitioned into training and test subsets by assigning 5 fragments for training and 4 for testing. Test fragments were selected to represent a cohesive ${4}\,\upmu \hbox {m} \times {4}\,\upmu \hbox {m}$ surface patch at the lower right corner of the fully stitched image. Table 1 shows the total number of annotated defects (*N*) and average defect density ($N_{def}$) for each AFM image cell and training/test subset. Defect density is expressed as the number of defects per square micrometer.

**Figure 1:**
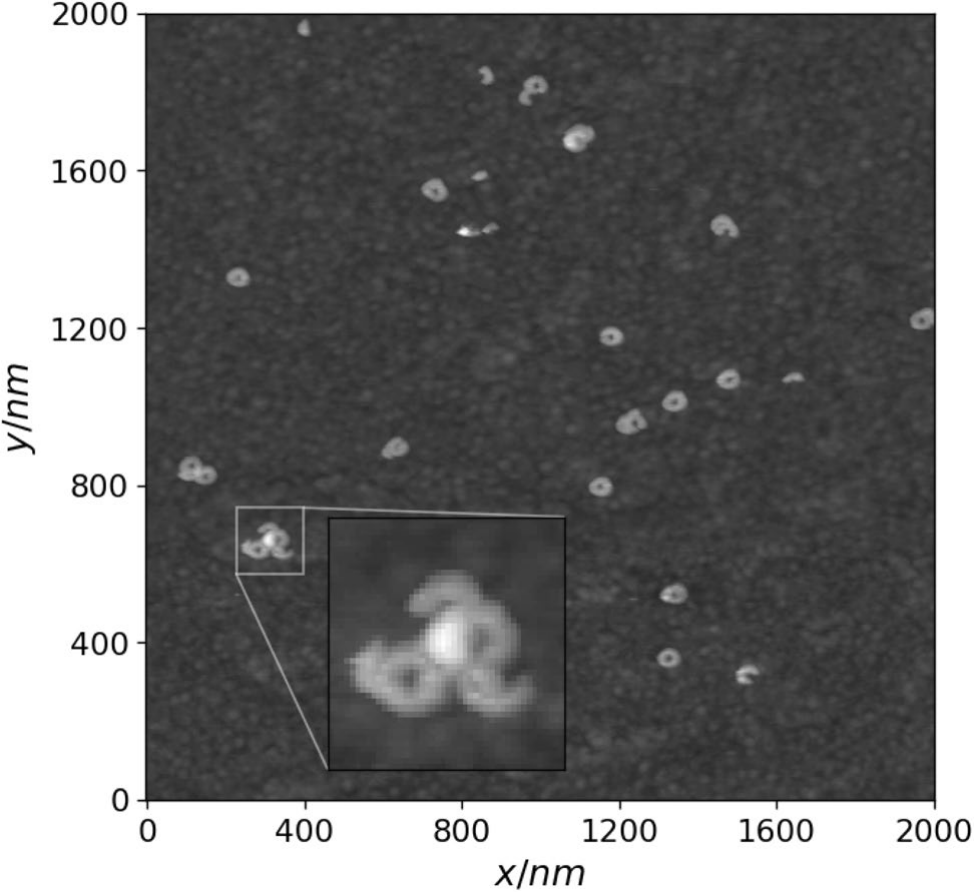
Example of an AFM image fragment with an instance of defect cluster zoomed in.

In addition to aforementioned parameters each surface image is also characterized by metric $\sigma$ which is obtained by computing the Voronoi diagram for a given defect set and calculating the standard deviation of the normalized Voronoi sector areas (multiplied by defect density $N_{def}$). This quantity summarizes the degree of defect clustering where higher values correspond to stronger clustering effect (example of defect cluster is highlighted in Fig. 1). Defect clustering has been shown to have significant influence on EIS spectra of tBLM membranes, as presented in earlier research^10^.

**Table 1:** AFM image sets used for defect detection model training and testing.

AFM surface	Subset	Image fragments	*N*	$N_{def}$	$\sigma$
1	Training	5	202	10.10	1.18
2	Training	5	138	6.90	1.12
3	Training	5	170	8.50	0.77
1	Test	4	172	10.75	1.20
2	Test	4	97	6.06	1.02
3	Test	4	158	9.88	0.91

### Defect detection accuracy

Although membrane defects are primarily characterized by their center coordinates and defect radius, these attributes can be used to express the defect position in the image as its bounding rectangle. By comparing two sets of bounding rectangles, corresponding to true and predicted defect positions, defect detection accuracy can be quantitatively evaluated.

**Figure 2:**
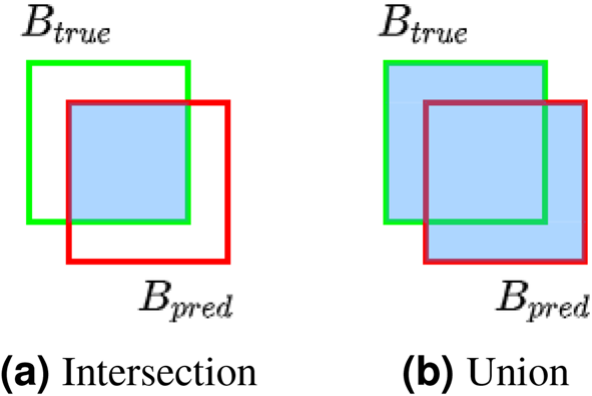
Bounding rectangle overlap of true and predicted defect positions.

To count the number of correct detections, the bounding rectangle of each true defect position ($B_{true}$) is matched with its closest prediction ($B_{pred}$). The overlap between each such pair of true and predicted bounding rectangles is evaluated by the intersection over union (*IoU*) metric (1) (also known as Jaccard index), which is expressed as the ratio of bounding rectangle intersection and union areas (Fig. 2):

1 $$\begin{aligned} IoU = \frac{B_{true} \cap B_{pred}}{B_{true} \cup B_{pred}}. \end{aligned}$$

Higher *IoU* values correspond to a better match between both bounding rectangles. If *IoU* value is above the chosen threshold (i.e. 0.5), the detection is assumed to be a true positive (TP). Otherwise, if no matching prediction exists for a given true position, such detection is counted as a false negative (FN). In the opposite case, when no true bounding rectangle can be matched for a given prediction, a false positive (FP) is assumed. By counting all such cases of correct and incorrect detections, overall defect detection accuracy is summarized by precision and recall metrics^24^:

2 $$\begin{aligned} Precision= & {} \frac{TP}{TP + FP}. \end{aligned}$$

3 $$\begin{aligned} Recall= & {} \frac{TP}{TP + FN}. \end{aligned}$$

Both precision and recall can also be expressed by the *F*1 metric:

4 $$\begin{aligned} F1 = 2 \times \frac{Precision \times Recall}{Precision + Recall}. \end{aligned}$$

### Synthetic defect set generation

In order to assess the relationship between defect detection accuracy and corresponding variations in EIS spectra, a substantial number of defect detection result sets is required. Such detection results should exhibit different precision and recall values distributed in a certain range. However, such specific detection results can be difficult to acquire by applying object detection models trained using real AFM images and annotated true defect positions. We chose an alternative approach of synthetically generating defect coordinate sets which would emulate defect detection results at different accuracy levels. Each synthetic case is generated by starting with the initial set of known true defect coordinates and applying certain modifications (defect addition, removal, coordinate shifting) to acquire a new defect set equivalent to the defects actually being detected by some model with imperfect accuracy.

**Figure 3:**
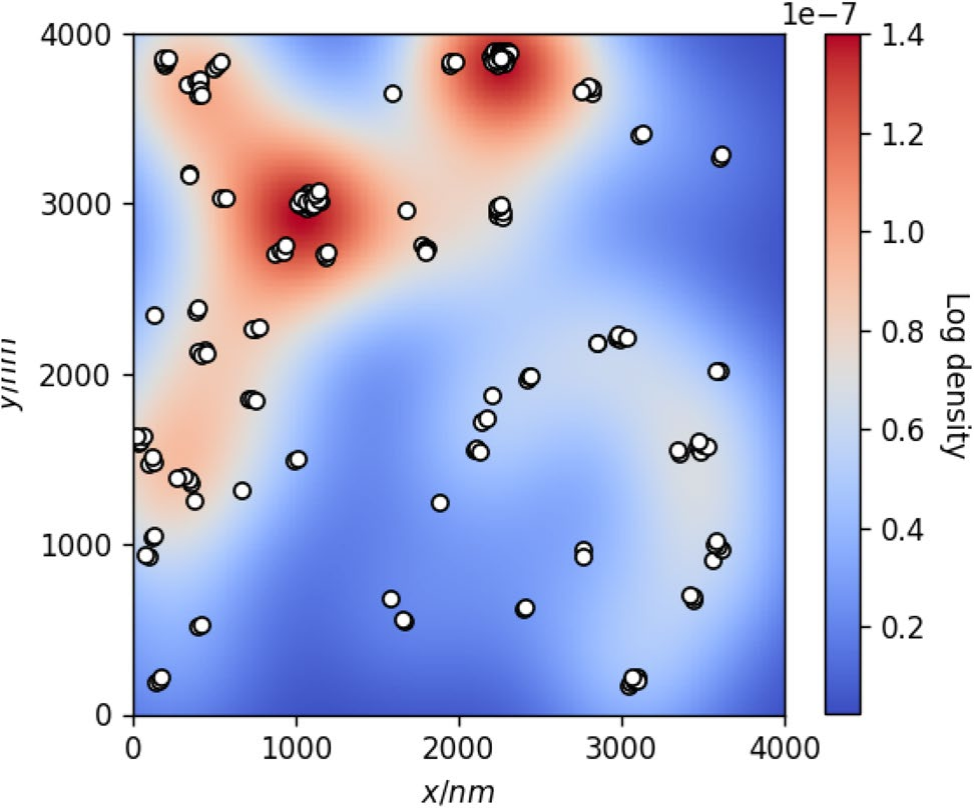
KDE model of true defect coordinates (white dots) annotated for AFM test image #1. Background color represents the log probability density of the fitted KDE distribution.

The procedure for generating a series of such synthetic cases from a given true defect set consists of the following steps:

1. Kernel density estimation (KDE)^25^ is applied for the set of true defect coordinates. The resulting distribution is used to reduce the chances of defect clustering changing significantly due to new defects being added or existing ones removed. Figure 3 shows an example of a clustered defect set and its corresponding KDE distribution, where warmer colors correspond to the higher values of its probability density function.
2. For each synthetic case:
  - True coordinates ($x^{(true)}$ and $y^{(true)}$) of each existing defect are modified by adding normally-distributed random values:
    $$\begin{aligned} x^{(pred)} = x^{(true)} + \delta ; \qquad y^{(pred)} = y^{(true)} + \delta ; \qquad \delta \sim {\mathscr {N}}(0, s^2) \end{aligned}$$
    This results in realistically imperfect matches between true and predicted bounding rectangles of the defects.
  - A number $n_{remove}$ of defect coordinate pairs are sampled from the KDE distribution. True defects closest to the sampled coordinates are selected and removed from the initial defect set. This introduces false negatives (FN) into the generated defect set and reduces recall accordingly.
  - A number $n_{add}$ of new coordinate pairs are sampled from the KDE distribution and defects with these coordinates are added into the generated defect set. This represents false positives (FP) and corresponds to lowered precision values.

**Figure 4:**
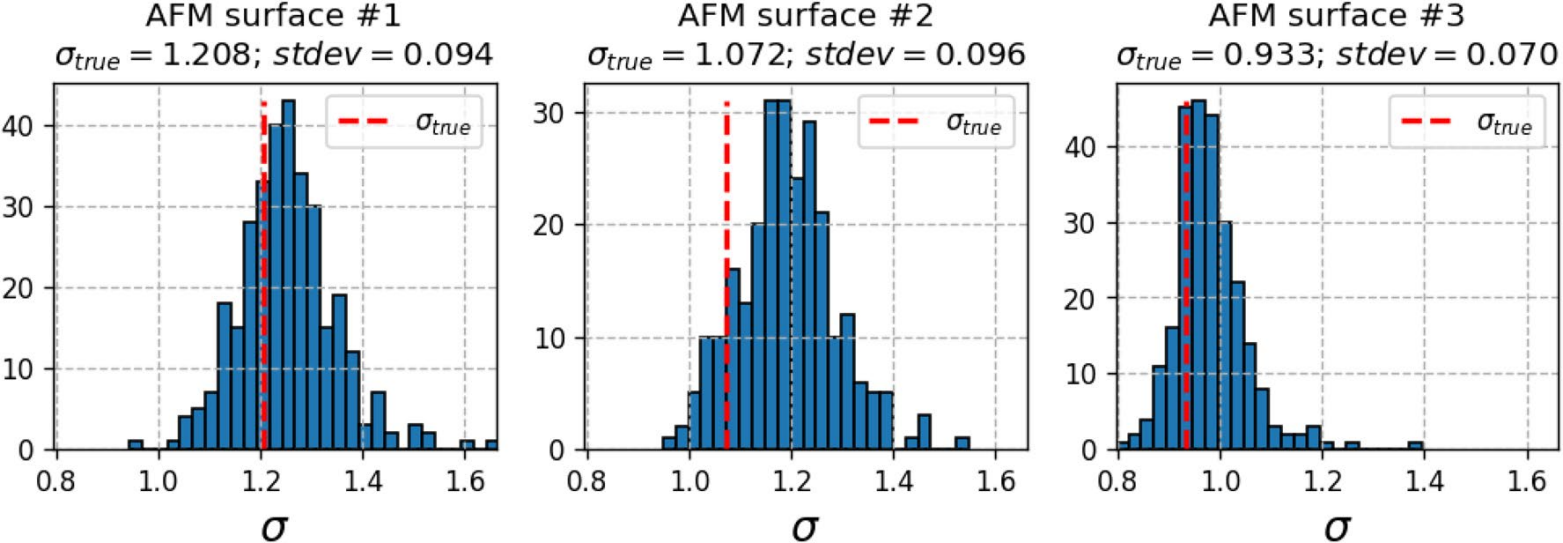
Histograms of standard deviations ($\sigma$) of normalized Voronoi sector areas (multiplied by defect density $N_{def}$) computed for synthetically generated defect distributions. Red dashed lines—Voronoi sector areas of true defect distributions.

The described algorithm was used to generate the synthetic cases for each of three AFM test images independently. KDE distributions were fitted using the Gaussian kernel and bandwidth parameter set to 400. The standard deviation parameter *s* of the normal distribution used for defect coordinate shifts was set to 4. Parameters $n_{remove}$ and $n_{add}$ were initially set to 0 and then incremented throughout the generation process by a step quantity corresponding to 3% of true defect count *N* until the maximum value of *N*/2 was reached. Table 2 shows the properties of the synthetic defect sets generated by the described procedure. Due to stochastic nature of this algorithm, some variability of clustering effect (expressed in terms of $\sigma$) is still present in the defect sets, as summarized in Fig. 4.

**Table 2:** Summary of generated defect sets for each AFM test image. Precision, recall and F1 values were computed against true defect sets annotated in the given AFM image.

AFM surface	Generated cases	Precision range	Recall range	F1 range
1	324	0.49 – 1.00	0.48 – 0.99	0.49 – 0.98
2	256	0.54 – 1.00	0.51 – 1.00	0.53 – 1.00
3	256	0.53 – 1.00	0.51 – 1.00	0.53 – 0.99

### EIS modeling

**Figure 5:**
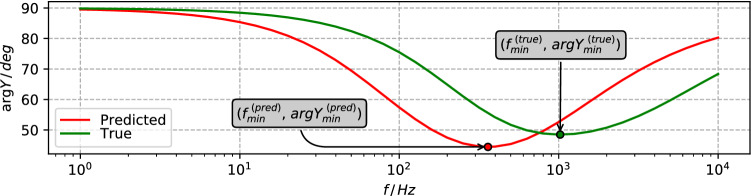
Spectral features of modeled EIS spectra involved in quantifying the difference between true and predicted defect set cases.

Electrochemical impedance (EIS) spectra of each defect distribution are modeled by applying the finite element analysis (FEA) technique. Membrane models were implemented and solved in the same way as described in the previous study^9^. Modeling was performed for each AFM surface from the test set by using the true defect distribution and each of the generated cases, described in “Synthetic defect set generation” and referred to as the predicted set. In order to quantify the discrepancy between the EIS spectra modeled for any given pair of true and predicted defect sets we used the positions of the minima points of the curves (example in Fig. 5) along both frequency and admittance phase axes:

5 $$\begin{aligned} \mathrm {\Delta \,f_{log}}= & {} \log _{10}(f_{min}^{\,(true)}) - \log _{10}(f_{min}^{\,(pred)}) \end{aligned}$$

6 $$\begin{aligned} \Delta \arg Y= & {} \arg Y_{min}^{(true)} - \arg Y_{min}^{(pred)} \end{aligned}$$

In order to characterize the relationship between the defect detection accuracy and deviations in the resulting EIS spectra, using F1 metric alone is not enough due to the fact that EIS spectral features are more strongly influenced by the defect size and density than by the specific positions of the defects in the membrane surface^9^. For this reason, a predicted defect set might poorly match the true one and thus exhibit a low F1 value, although their corresponding EIS spectra might closely match, as long as the overall properties of defect count and size are similar. To take this effect into account we also use an additional $Q_N$ metric which represents the ratio of defect densities (number of defects per square micrometer) from predicted and true defect sets:

7 $$\begin{aligned} {\mathrm {Q}}_{\mathrm {N}} = N_{def}^{(pred)} / N_{def}^{(true)} \end{aligned}$$

## Results and discussion

### Defect detection with convolutional neural network

To perform the actual defect detection experiments using AFM image data a convolutional neural network (CNN) model was chosen as the current state-of-the-art approach for object detection tasks. Specifically, we used a popular SSD FPN architecture object detector^26^ implementing a two-stage object detection approach, where the candidate locations of objects are first identified and then each region is classified separately. Initial model^27^ was pre-trained with COCO image dataset^28^ to detect objects of 90 different types. In order to adapt it for defect detection in AFM images, the model was re-trained to detect a single type of object (membrane defect) using 15 AFM images described in Table 1 and containing a total of 510 annotated defect instances. Each training image fragment with $512 \times 512$ resolution was scaled to match the model input of $640 \times 640$ color (RGB) images. Tensorflow 2.0 framework was used to train and evaluate the model and the training was performed using Nvidia GTX 1080 GPU hardware.

The trained model was evaluated with each of 12 test image fragments (Table 1) and the detection results were aggregated to match the layout of 4 stitched fragments per each AFM surface. Bounding boxes of all detected defect instances were equalized to match the width and height of 50 nm, corresponding to defects with circular radius of 25 nm. Defect instances predicted by the model were compared with the true defect positions and the overall model accuracy was evaluated using the precision, recall and F1 metrics for each AFM surface (Table 3).

**Figure 6:**
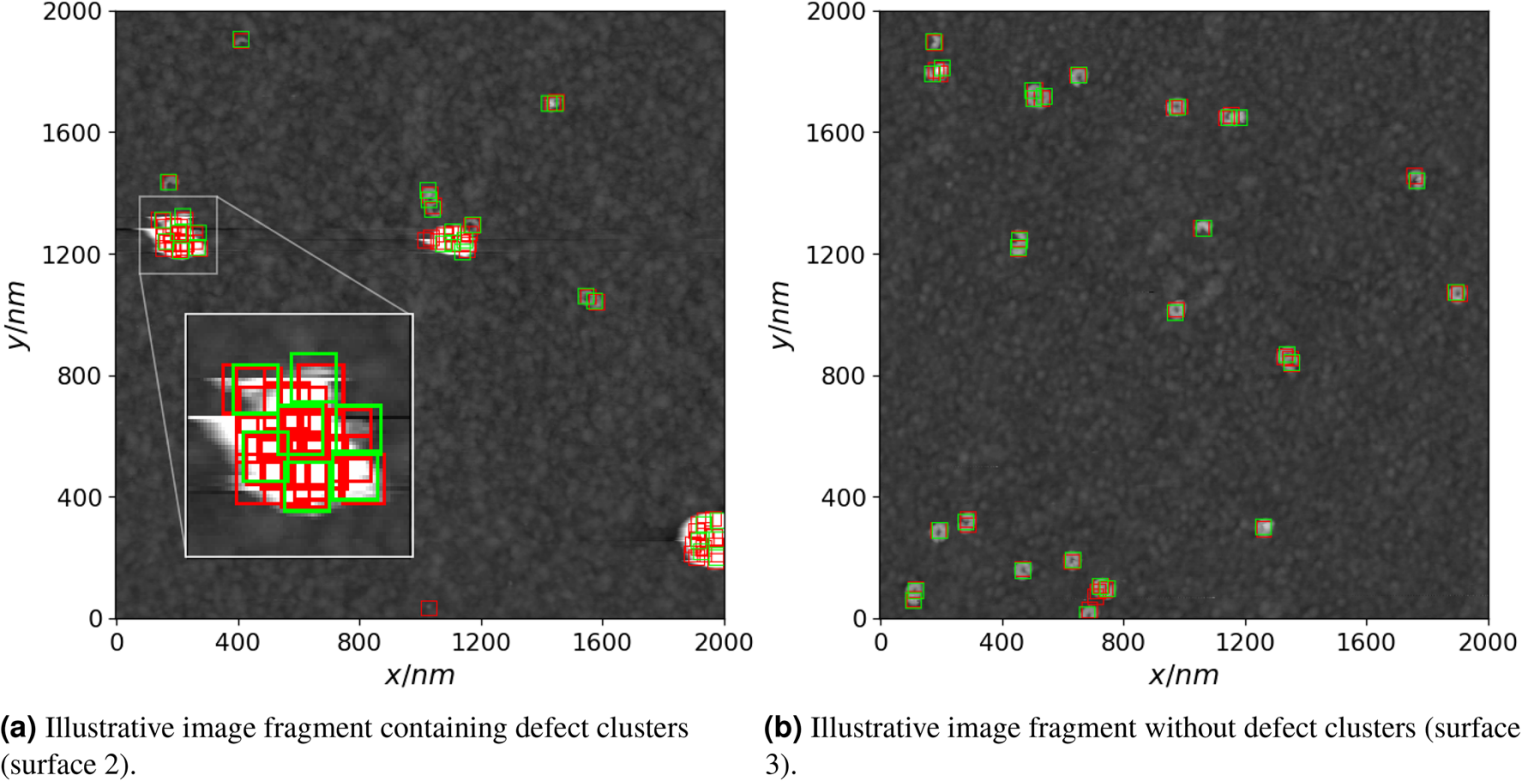
Examples of true defect positions (green rectangles) and predicted (red rectangles) ones by using the convolutional neural network. An instance of a defect cluster and the corresponding true and predicted defect positions is zoomed in on the left image.

Precision, recall and F1 scores indicate a significant number of inaccurate detections in the test images of all three AFM surfaces. Defect clusters (Fig. 6, left) proved to be difficult to resolve due to poorly visible surface features inside the clusters. However, the model performed fairly well for certain image fragments with no defect clusters present (Fig. 6, right). This is also illustrated by the fact that the test image of AFM surface 3 which indicates the lowest amount of defect clustering in terms of $\sigma$ (Table 1) also have the highest overall F1 score.

**Table 3:** Defect detection accuracy of the test AFM images and the resulting differences between EIS spectra of true and predicted defect sets.

AFM surface	Precision	Recall	F1	$\mathrm {Q}_\mathrm {N}$	$\Delta \,\mathrm {f}_{\mathrm {log}}$	$\Delta \arg \mathrm {Y}$
1	0.775	0.581	0.664	0.750	0.009	0.735
2	0.555	0.680	0.611	1.227	$-0.013$	0.681
3	0.757	0.728	0.742	0.962	$-0.027$	0.864

### How much inaccuracies in detection of defects affect the prediction of EIS response of tBLMs?

As seen from the previous paragraph, the current AI-based algorithm has limited precision of detection of defects in real AFM pictures. Specifically, as seen from Table 3, both parameter F1, and number of entities $\mathrm {Q_N}$ are detected with max 75% (*F*1) and max 96% ($\mathrm {Q_N}$) precision as judged from the tests on surfaces 1, 2 and 3 (Table 3). It is however, important if inaccuracy in defect recognition can result in significant deviations in predictive power of EIS spectral features. To answer this question we compared the position of characteristic points of EIS spectra obtained via FEA modeling of EIS curves based on coordinates determined by eye (“true coordinates”) and EIS curves obtained by applying the AI algorithm. The comparison of the curves are performed by calculating the position of the EIS Bode admittance phase curve minimum in the $\arg Y$ vs $\log f$ plane. The deviation along the $\log f$ axis is measured on a logarithmic scale as $\mathrm {\Delta \,f_{log}}$ and the deviation along the $\arg Y$ axis is measured on a linear scale as $\Delta \arg Y$. Table 3 summarizes the findings. It is obvious that the shift of the position of the phase minima is within the approximate interval 0.1 and -0.027, which translates into the range for relative error in the position of the minimum on a $\log f$ scale from 2 to 6%. Even though modern EIS workstations provide much greater measurement precision, given limitations related to the reproducibility of a specific tBLM experiment such error may be considered as acceptable. The position of the phase minimum on the $\log f$ scale is a main parameter from which the defect density can be estimated from the EIS spectra^9,13,14^. So, from this series of tests we may hypothesize that the precision of the prediction of defect density using AI-based algorithm can be increased by recalculating the defect density from the AI-algorithm predicted position of the $\log f_{min}$ using previously described method^9^. For example, in sample 2, the AI-derived QN is 1.227, i.e, 22.7% more than is located in real AFM images. However, the $\mathrm {\Delta \,f_{log}}$ shift is only -0.013, which translates into -3% with respect to a true defect density value.

This result is of upmost importance because it suggests that the AI-based AFM image analysis allows to reconstruct EIS spectra with satisfactory precision, while combination of both theoretical analysis techniques, EIS^9^ and AI-based AFM image analysis allows to precisely determine defect densities on real tBLM samples.

### Simulation of inaccuracies in detection of defects in tBLMs

**Figure 7:**
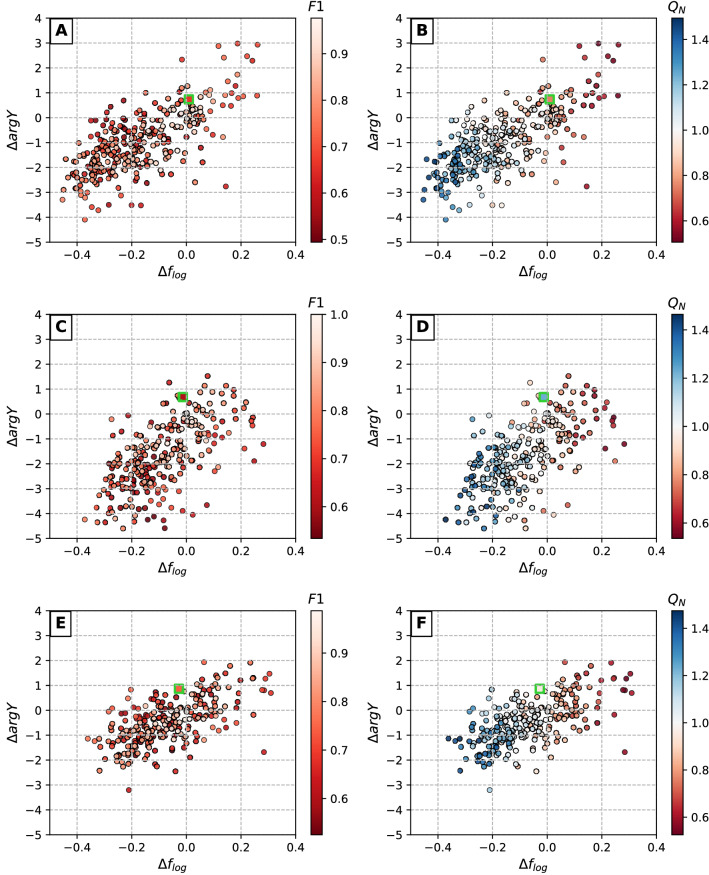
Dependencies between defect detection accuracy (expressed in terms of *F*1 and $Q_N$) and deviations in corresponding EIS spectra. Coloured dots represent synthetically generated defect sets at varying detection accuracy levels (Table 2), squares with green borders indicate real detection results obtained with CNN model (Table 3). Scatter plot pairs A/B, C/D and E/F represent AFM surfaces 1, 2 and 3 respectively.

In the previous paragraph the evaluation analysis of the AI-based AFM data analysis algorithm was evaluated using images of 3 real samples. To obtain statistically more significant estimate of how the precision of AI-based algorithm may affect the prediction of the EIS spectral features we applied simulation of the inaccuracies in defect coordinate detection. This was done as described in “Synthetic defect set generation” . Starting with true distribution we aimed at generating a large number of defect distributions and determine deviations from true distributions which may arise due to lack of precision of AI-based defect detection algorithm. The simulation data is summarized graphically in Fig. 7. Green points in Fig. 7 plots correspond to the positions of characteristic points of samples 1, 2, and 3, which are included in Table 3.

As seen from Fig. 7 deviation of parameter $F1 < 1$ results in skewed dispersion of both parameters $\mathrm {\Delta \,f_{log}}$ and $\mathrm {\Delta \arg Y}$ (see Supplemental Material). Such asymmetry of parameter distribution introduces a systemic shift of $\mathrm {\Delta \,f_{log}}$ in AI-derived AFM image data, which for samples 1, 2, and 3 were found to be $-0.168, -0.083$ and $-0.068$ respectively (see Supplemental Material) in the F1 values interval from 0.5 to 1.0. The standard deviations of parameter $\mathrm {\Delta \,f_{log}}$ are 0.16, 0.13 and 0.14 for samples 1, 2 and 3 correspondingly (F1 interval [0.5,1.0]). Relatively small, though consistent shift of $\mathrm {\Delta \arg Y}$ was also detected. Specifically, the following shifts were observed for samples 1, 2 and 3 respectively: −0.98 deg, −1.68 deg and −0.48 deg in the same F1 interval. The systematic shifts $\mathrm {\Delta \,f_{log}}$ decrease rapidly as F1 approaches 1. The $\mathrm {\Delta \,f_{log}}$ and its standard deviation for F1 interval from 0.95 to 1.0 are 0.001 and 0.027, −0.002 and 0.031, and −0.016 and 0.027 for samples 1, 2 and 3 respectively.

Currently, we cannot provide any reasonable explanation for such negative shift. It is obvious that the systemic negative shift may vary in relatively wide intervals causing errors in predictions of EIS spectra features. We may state that the precision of AI-based algorithm reflected in parameter F1 may considerably affect the position of $f_{min}$ so that the relative errors in predicting this parameter may exceed several tens of percent. In our sample surfaces 1, 2 and 3 the F1 values 0.664, 0.611 and 0.742 resulted in (see Supplemental Material Tables S2, S3 and S4, left panes) systemic shifts of $\mathrm {\Delta \,f_{log}}$ $-0.174, -0.070$ and $-0.073$ respectively.

### Predicting physical parameters of tBLMs from comparison of AFM image derived and experimentally measured EIS spectra

The comparison of AFM derived and experimentally measured EIS spectra allows one to make estimates of some important physical parameters of tBLMs. Specifically, the specific resistance, $\rho$, of submembrane layer separating phospholipid bilayer and metal/solution interface (Helmholtz layer) can be estimated. This parameter cannot be independently estimated from the analysis of the EIS response, because it is fully correlated with the defect density $N_{def}$^13^. Independent estimation of $N_{def}$ using AI-based AFM image analysis algorithm allows to resolve the uncertainty. In such exercise the range of defect radius can also be estimated because $r_{def}$ determines the position of the phase minimum of $\arg Y$ vs. $\log f$ plot of EIS spectra of tBLMs.

**Figure 8:**
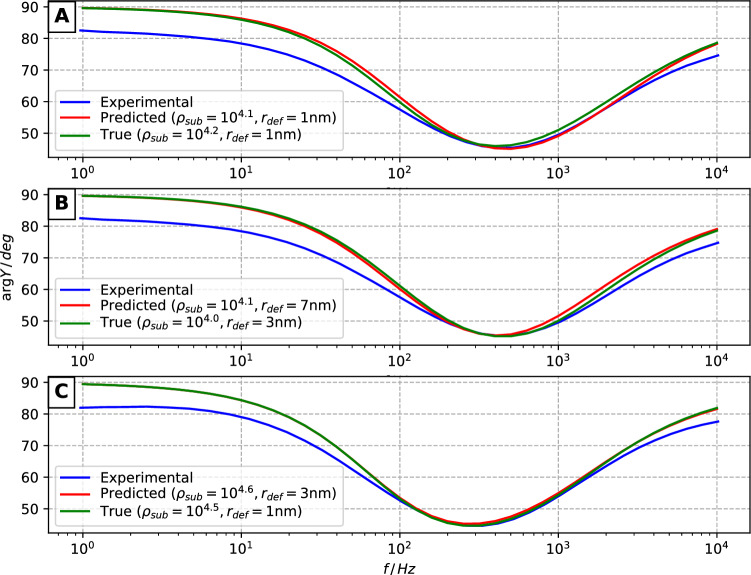
Admittance phase data of experimental EIS measurements (blue curves) versus modeled cases (green and red curves corresponding to manually annotated defect coordinates and CNN model predictions, respectively). **(A–C)** correspond to AFM surfaces 1, 2 and 3.

A series of FEA modeling tasks were performed with each pair of true (established by eye) and predicted defect sets for all three AFM surfaces (test data) separately. Two parameters were varied in each scenario: defect radius $r_{def}$ was adjusted from 1 nm to 13 nm with increments of 2 nm, while the specific conductivity of the submembrane layer $\rho _{sub}$ was adjusted in logarithmic scale from $10^{4}$ to $10^{5}$ $\Omega \cdot \hbox {cm}$ with power increments of 0.1, resulting in a total of 77 parameter combinations. Modeled curves of both true and AI-predicted defect sets were matched against the experimental EIS data by minimizing the L1 norm of minimum point coordinates (frequency and admittance phase axes) between a pair of curves. Figure 8 shows the modeled and experimental curves of each surface as well as the specific $r_{def}$ and $\rho _{sub}$ values of the corresponding modeled cases.

The mean $r_{def}$ and $\rho _{sub}$ values were found to span interval from 1 to 7 nm and $10^{4.0}$ to $10^{4.6}$ $\Omega \cdot cm$ correspondingly. The mean values of the parameters are correspondingly $2.7 \pm 1.0$ nm and $10^{4.25 \pm 0.10}$ $\Omega \cdot cm$. While $r_{def}$ shows significant standard deviation, which is expected because sensitivity of EIS response to $r_{def}$ is small if relatively modest interval of $r_{def}$ variation is considered^13^. In opposite, $\rho _{sub}$ can be established with considerably better precision, so it is likely that the described AI-based AFM image analysis technique has a good perspective for the use in calibration of tBLMs systems for the precision measurement of defect densities which is of upmost importance in considering tBLMs as quantitative biosensors for the detection of pore-forming toxins.

## Conclusions

In this study we investigated the possibilities of automated detection of defects in AFM images of tBLM membranes and possibilities to predict the EIS response of such membranes. By applying the convolutional neural network for the formulated object detection task we demonstrated the potential advantage of this approach in comparison to manual defect annotation, although the results should be considered as preliminary due to the limited amount of image data used and no model tuning.

We also attempted to solve the defect detection problem by using TopoStats automated biomolecule tracing tool^23^ and compared its accuracy to the performance of the CNN approach (see Supplemental material, Table 5S). The precision of TopoStats proved to be comparable to CNN, while the recall was significantly lower for all AFM images, indicating that a large portion of actual defects were not detected by the tool (illustrative examples presented in Supplemental Material, Fig. 1S). Poor performance of TopoStats can be attributed to the presence of defect clusters in the images. This proves to be a significant obstacle for object detection approaches based on non-AI image processing methods.

Using three different samples of tBLMs we found that true and AI-derived sets of defect coordinates though being non-identical produce by FEA modeling similar EIS curves. One of the main EIS spectral features, the predicted position of the phase minimum in Bode plots of admittance was within 2–6% from the true values.

Test on larger sample sets, which coordinates were produced synthetically, indicate possibility of a systematic deviations of predicted EIS spectral features. These deviations are sensitive to the AI algorithm’s precision parameter F1, and they rapidly decrease as F1 approaches 1. Taken together these findings show that EIS spectra can be predicted sufficiently well however, the systematic errors need to be taken into account.

We also showed that automated AI-based algorithm of AFM image analysis allows one to make EIS spectra predictions which can be used to assess important physical parameters of tBLMs such as submembrane specific resistance. Using three different samples of tBLMs we found that the submembrane resistance is $10^{4.25 \pm 0.10}$ $\Omega \cdot cm$, a value slightly lower compared to value previously used ($10^{4.5}$ $\Omega \cdot cm$). This parameters allows calibration of tBLM biosensors for quantitative detection of activities of pore-forming toxins.

In conclusion we provide evidence of applicability of AFM to assess the geometry and density of membrane damaging defects such as pore-forming toxins in tBLMs. This data can be used to theoretically predict EIS response of tBLMs as well as calibrate this response for biosensor applications.

## Supplementary Information

Supplementary Information.

## Data Availability

Modelling data and experimental data are available from authors on request.
