# Supplementary material for: AI-based atomic force microscopy image analysis allows to predict electrochemical impedance spectra of defects in tethered bilayer membranes
Source: Sci Rep. 2022 Jan 21;12:1127. doi: 10.1038/s41598-022-04853-4 (PMC8783026; doi:10.1038/s41598-022-04853-4)
Supplement: Supplementary file 1 — Supplementary Information. [file 41598_2022_4853_MOESM1_ESM.pdf]

AI-based Atomic Force Microscopy Image Analysis Allows to Predict Electrochemical Impedance Spectra of Defects in Tethered Bilayer Membranes: Supplemental Material

Tomas Raila[1], Tadas Penkauskas[2], Filipas Ambrulevičius[2], Marija Jankunec[2], Tadas Meškauskas[1], and Gintaras Valinčius[2,*]

[1]Institute of Computer Science, Vilnius University, Didlaukio 47, LT-08303 Vilnius, Lithuania
[2]Institute of Biochemistry, Life Sciences Center, Vilnius University, Saulėtekio 7, LT-10257 Vilnius, Lithuania
[*]gintaras.valincius@gmc.vu.lt

1 Dependency between defect detection accuracy and differences in EIS spectra

The following tables summarize the spectral deviations between real and predicted defect sets (expressed by Δf_{log} and $\Delta \arg Y$) synthetically generated for each AFM surface. Table 1S presents the overall statistics of the generated defect set series, while the tables 2S - 4S show these properties at discrete detection accuracy levels represented in terms of $F1$ and Q_N.

Table 1S. Summary statistics of defect sets generated for each AFM surface

AFM surface	Δf_{log}				ΔargY			
	Mean	Median	Stdev	Skewness	Mean	Median	Stdev	Skewness
1	-0.168	-0.191	0.162	0.484	-0.977	-1.090	1.242	0.443
2	-0.083	-0.090	0.132	0.386	-1.683	-1.729	1.330	-0.003
3	-0.068	-0.087	0.140	0.508	-0.485	-0.498	0.904	0.165

Table 2S. AFM surface 1

F1	Δf_{log}		ΔargY	
	Mean	Stdev	Mean	Stdev
0.50 – 0.55	-0.240	0.078	-0.969	0.804
0.55 – 0.60	-0.220	0.092	-1.095	1.121
0.60 – 0.65	-0.174	0.165	-0.959	1.274
0.65 – 0.70	-0.115	0.199	-0.587	1.609
0.70 – 0.75	-0.167	0.178	-1.056	1.525
0.75 – 0.80	-0.221	0.175	-1.317	1.200
0.80 – 0.85	-0.188	0.151	-1.114	0.954
0.85 – 0.90	-0.126	0.111	-0.870	0.926
0.90 – 0.95	-0.057	0.065	-0.517	0.825
0.95 – 1.00	0.001	0.027	-0.113	0.196

Q_N	Δf_{log}		ΔargY	
	Mean	Stdev	Mean	Stdev
0.5 – 0.6	0.197	0.049	1.421	1.757
0.6 – 0.7	0.092	0.075	0.533	1.058
0.7 – 0.8	-0.012	0.079	-0.112	0.975
0.8 – 0.9	-0.081	0.090	-0.588	1.148
0.9 – 1.0	-0.160	0.100	-0.720	0.835
1.0 – 1.1	-0.203	0.091	-1.150	0.770
1.1 – 1.2	-0.264	0.068	-1.610	0.655
1.2 – 1.3	-0.314	0.053	-2.008	0.719
1.3 – 1.4	-0.365	0.029	-2.148	0.762
1.4 – 1.5	-0.419	0.060	-2.270	0.632

Table 3S. AFM surface 2

F1	Δf_{log}		ΔargY	
	Mean	Stdev	Mean	Stdev
0.50 – 0.55	-0.130	0.016	-4.183	0.089
0.55 – 0.60	-0.170	0.065	-2.437	1.396
0.60 – 0.65	-0.070	0.128	-2.049	1.203
0.65 – 0.70	-0.085	0.149	-1.490	1.468
0.70 – 0.75	-0.069	0.153	-1.849	1.316
0.75 – 0.80	-0.106	0.143	-1.704	1.335
0.80 – 0.85	-0.091	0.137	-1.810	1.309
0.85 – 0.90	-0.077	0.105	-1.234	1.126
0.90 – 0.95	-0.032	0.075	-0.936	0.775
0.95 – 1.00	-0.002	0.031	-0.235	0.481

Q_N	Δf_{log}		ΔargY	
	Mean	Stdev	Mean	Stdev
0.5 – 0.6	0.205	0.072	-0.506	0.779
0.6 – 0.7	0.127	0.077	-0.035	0.755
0.7 – 0.8	0.061	0.073	-0.825	1.544
0.8 – 0.9	-0.014	0.078	-1.045	1.025
0.9 – 1.0	-0.072	0.083	-1.757	1.340
1.0 – 1.1	-0.121	0.080	-1.900	1.031
1.1 – 1.2	-0.169	0.052	-1.989	0.794
1.2 – 1.3	-0.222	0.051	-2.556	1.000
1.3 – 1.4	-0.235	0.055	-2.921	1.056
1.4 – 1.5	-0.294	0.051	-3.457	0.628

Table 4S. AFM surface 3

F1	Δf_{log}		ΔargY	
	Mean	Stdev	Mean	Stdev
0.50 – 0.55	-0.110	0.063	-1.087	0.735
0.55 – 0.60	-0.100	0.088	-0.363	0.527
0.60 – 0.65	-0.030	0.142	-0.371	0.969
0.65 – 0.70	-0.032	0.166	-0.442	1.063
0.70 – 0.75	-0.073	0.165	-0.391	1.092
0.75 – 0.80	-0.091	0.163	-0.522	1.008
0.80 – 0.85	-0.092	0.129	-0.777	0.827
0.85 – 0.90	-0.061	0.109	-0.282	0.746
0.90 – 0.95	-0.046	0.080	-0.414	0.456
0.95 – 1.00	-0.016	0.027	-0.334	0.329

Q_N	Δf_{log}		ΔargY	
	Mean	Stdev	Mean	Stdev
0.5 – 0.6	0.270	0.044	0.362	1.214
0.6 – 0.7	0.180	0.071	0.869	0.829
0.7 – 0.8	0.090	0.043	0.255	0.696
0.8 – 0.9	0.023	0.063	-0.205	0.849
0.9 – 1.0	-0.062	0.054	-0.426	0.640
1.0 – 1.1	-0.107	0.054	-0.614	0.593
1.1 – 1.2	-0.175	0.046	-0.968	0.752
1.2 – 1.3	-0.218	0.040	-1.101	0.657
1.3 – 1.4	-0.226	0.048	-1.357	0.509
1.4 – 1.5	-0.306	0.045	-1.538	0.554

2 Defect detection with TopoStats

Table 5S shows the results of automated defect detection in raw AFM images which was performed by using TopoStats automated biomolecule tracing tool. The default parameter settings specified for membrane attack complex (MAC) pore detection were adjusted by setting the minimum area to 2×10^{-7} and the maximum and minimum deviation from the median pixel size to 5.0 and 0.5 respectively. Each detected grain was treated as an individual defect with its coordinates derived from the center of the grain area. Precision, recall, F1 and Q_N values were computed as described in section 2 of the article. Figure 1S shows visual examples of defect positions obtained by TopoStats compared with true defect positions (manually annotated by domain expert).

Table 5S. Defect detection (with TopoStats) accuracy in test AFM images.

AFM surface	Precision	Recall	F1	Q_N
1	0.754	0.233	0.355	0.308
2	0.742	0.237	0.359	0.319
3	0.886	0.443	0.591	0.500

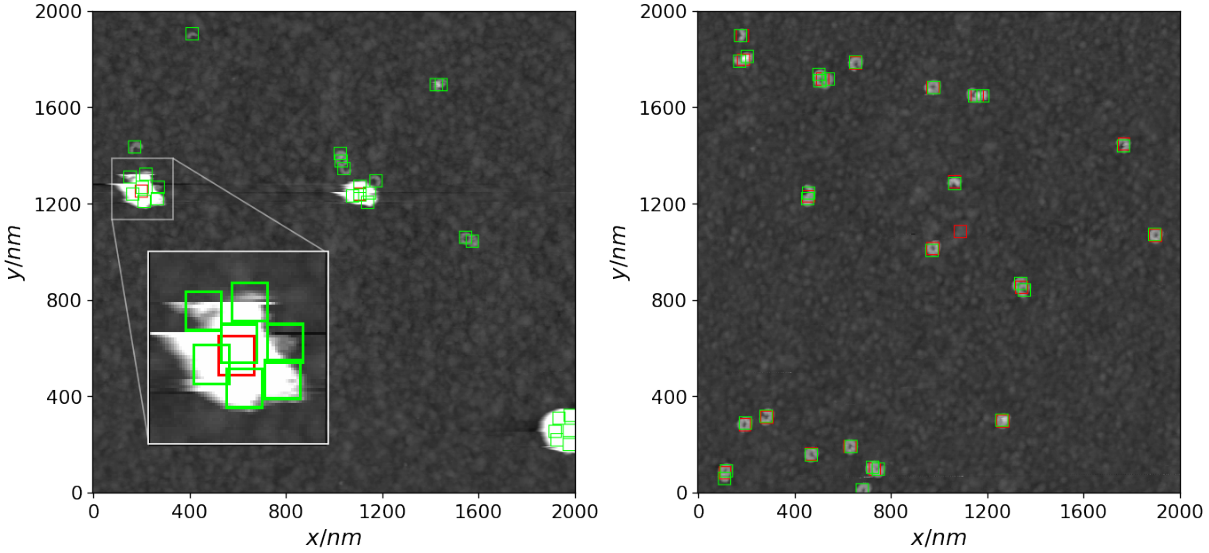

(a) Illustrative image fragment containing defect clusters (surface 2).

(b) Illustrative image fragment without defect clusters (surface 3).

Figure 1S. Examples of true defect positions (green rectangles) and detected ones by using TopoStats tool (red rectangles). An instance of a defect cluster and the corresponding true and predicted defect positions is zoomed in on the left image.
